# The thin space between individuals and contexts as affordance for healthy longevity: a psychological perspective for aging in place studies

**DOI:** 10.3389/fragi.2025.1632041

**Published:** 2025-10-16

**Authors:** Stefania Butti, Francesca Morganti

**Affiliations:** ^1^ Department of Human and Social Sciences, University of Bergamo, Bergamo, Italy; ^2^ Center for Healthy Longevity - University of Bergamo, Bergamo, Italy

**Keywords:** aging in place, healthy aging, affordance, healthy longevity, urban aging, AIP study methodology, urbanization

## Abstract

It seems likely that the growing number of older adults and increasing urbanization will be among the most significant demographic and societal trends in the near future. These two global phenomena will undoubtedly have a profound effect on the demographic and geographical makeup of our world. In view of these changes, it is crucial that the health and social sciences consider how the concept of *Aging in Place* could play a valuable role in longevity studies. Considering this topic as correlated to different important themes such as functional, symbolic, and emotional attachment and importance of homes, neighborhoods, and communities - resumed in the categories of people, place and time - we introduce a new perspective in *Aging in (urban) Place* studies from a psychological perspective based on situated and embodied cognition, with the purpose of deeply analyzing the *thin space* between people and their context, viewing place not as a neutral backdrop but as a continuous opportunity for individuals to act. Only through an analysis of urban spaces as limits or possibilities in everyday life can we grasp how the city can be an adequate place to empower individuals’ healthy longevity.

## Aging and urbanization: two challenges of the nearest tomorrow

According to the World Health Organization (2023), the proportion of the world’s population aged over 60 will nearly double from 12% to 22% in the next few decades, highlighting one of the primary challenges of our century. Moreover, another emerging global phenomenon is considered to be the second major megatrend of the 21st century (United Nations, 2024): urbanization. In 2008, for the first time ever, the largest portion of people resided in cities, and the transition from rural to urban areas is expected to continue (UNFPA, 2007, as cited in Beard and Petitot, 2010), with urban settlements growing in number, land area, and population size across the globe (United Nations, 2019). This is not merely a migration, but a transformation of the built environment—hence why urbanization has been described as “one of the most powerful forces shaping the geography of the contemporary world” (Clark, 2000). It is profoundly changing the lifestyles of half of the world’s population (Michel, 2020; Smith, 2009). The intersection of these two global phenomena, population aging and increasing urbanization, requires the health and social science disciplines to question how the idea of urban aging can play an essential role in longevity studies and urban planning in the near future.

In light of the importance of the contexts of daily life and the demographic challenges described above, the World Health Organization (WHO) underlines the importance of studying the relationship between health and the urban environment (World Health Organization, 2017) to promote projects and beneficial practices to make cities “suitable for the old population” within shared perspectives of *urban health* and *active and healthy aging*.

The concept of active aging is often seen as a key point in this scenario. Active aging posits that older adults can maintain health and wellbeing by engaging in multiple domains of activity throughout later life (World Health Organization, 2002). It challenges deficit-oriented models by highlighting the competence, experience, and wisdom of older individuals rather than focusing solely on their limitations (Foster and Walker, 2021; Boudiny, 2013; Bowling and Dieppe, 2005). The concept emphasizes remaining active across social, economic, cultural, spiritual, and civic spheres, including physical activity and workforce participation (World Health Organization, 2002).

Although the concept of active aging is promoted by organizations such as the WHO, the European Union, and the United Nations as a “win-win” strategy for individuals and society, critical gerontology has raised significant objections. Critics argue that idealizing it may be counterproductive and even oppressive, and that policymakers often place excessive emphasis on physical activity while neglecting mental capacities, frequently equating it with simply working longer (Foster and Walker, 2021). Furthermore, some scholars criticize this paradigm for shifting the responsibility of “successful” aging onto individuals within the context of flexible capitalism and welfare retrenchment (Pfaller and Schweda, 2019). This fosters a “new ageism” that stigmatizes frailty (Van Dyk, 2014), imposes midlife values such as productivity and competitiveness, and denies the diversity of later life (Foster and Walker, 2015; Van Dyk, 2014).

Despite these critiques, active aging remains an important conceptual framework for understanding and responding to population aging (Foster and Walker, 2021), highlighting the interconnectedness of activity, health, independence, and wellbeing. It underscores the importance of maximizing opportunities for health, participation, and security to enhance quality of life throughout later life (World Health Organization, 2002), also starting from different aging in place solutions. The 2002 WHO report, *Active Aging: A Policy Framework*, delineates six determinants of active aging—*health and social service systems*, *behavioral factors*, *personal factors, physical environment*, *social environment*, and *economic factors*—and highlights their role in promoting age-friendly environments (World Health Organization, 2002). As noted by many studies (Siltanen et al., 2024; Bigonnesse and Chaudhury, 2022; Abdullah and Wolbring, 2013), aging well involves the dynamic use of resources and engagement in specific contexts. For this reason, it relates the theme of age-friendly cities, which foster active aging by adapting their structures and services to be inclusive and accessible to older individuals with different needs and capacities. A lack of such consideration could erode individuals’ wellbeing (OECD, 2025), a determining issue in the overall theme of aging in place. This will be discussed in the following paragraphs.

Since the mid-2000s, there has been growing interest in age-friendly issues (Buffel and Phillipson, 2024), leading to the launch of the WHO’s Global Age-Friendly City project in 2006, which currently involves 1739 cities and municipalities in 57 countries, encompassing over 370 million people worldwide (World Health Organization, 2025). The project aims to create urban environments that support the health, wellbeing, and participation of older adults. It focuses on eight domains considered important for individual wellbeing: *housing, transportation, respect and social inclusion, social participation, social and civic engagement, outdoor spaces and buildings, community support and health services,* and *communication and information* (World Health Organization, 2007).

As Buffel and Phillipson (2024) suggest, the program reflects the recognition of the critical role of both physical and social environments in upholding or enhancing the quality of life of older individuals and the influence of policies designed to support aging in place. This demonstrates that the phenomenon of aging extends beyond demographic trends and reveals its entanglement with spatial arrangements, social relations, and economic conditions (Yu, 2025).

As for the active aging approach, critical gerontology has emphasized that, by extension, aging in place risks being reframed as individual responsibilities, thereby generating new forms of exclusion (Van Dyk, 2014). This shift is closely linked to a neoliberal austerity logic that frequently transfers costs and responsibilities from the state to individuals and their support networks (Drilling et al., 2025), significantly shaping aging in place policies. Moreover, austerity, together with processes of globalization and urban regeneration, has reshaped urban environments in ways that often make neighborhoods hostile and challenging places for older people to age. These structural dynamics, in turn, directly affect the everyday experiences of older adults (Buffel et al., 2018).

## What we mean when we discuss aging in place

Scholars have identified aging in place as an achievable and worthwhile focus in efforts to integrate urban health and active aging (Bigonnesse and Chaudhury, 2020; Vasunilashorn et al., 2012). This is motivated by two different sets of influences. First, political movements related to older people aim to compensate for the welfare system, which may struggle to withstand the impact of aging populations (Pynoos et al., 2008). Second, it reflects people’s desire to continue living at home (see, e.g., Golant, 2020) and recognition of the importance of place for older people (McGrath and Hand, 2021). In response to these challenges, the concept has been adopted in public policy in many countries. For example, the United States (MetLife Mature Market Institute, 2010), the United Kingdom (Lofqvist et al., 2013; Hammarström and Torres, 2012; Sixsmith et al., 2014), and Australia (Stones and Gullifer, 2016) have adopted aging in place as a policy strategy to conserve resources (Greenberg and Schwarz, 2012). More recently, Australia has reformed the aged-care sector thanks to aging in place policies, moving away from the idea of expanding funding and service options. This framework aims to empower aged individuals by expanding their access to a wider range of support services, enabling them to stay in their homes if they wish to maintain their independence (Stones and Gullifer, 2016).

In other countries, we observed attempts to focus on housing-related issues. One example is the case of the UK, where a program has been developed to help older individuals maintain and improve their homes, enabling them to live independently at home for an extended period (Tinker, 1999). The rationale for enabling older people to live at home for longer is partly based on the belief that informal support from friends, neighbors, and families is more cost-effective than institutional care (Wiles et al., 2012). However, the relationship between aging in place and a country’s economic dynamics remains controversial. Several scholars have argued that it can reduce the costs of welfare policies, but there is still no empirical evidence to support this. Meanwhile, some scholars have expressed doubts about the real possibilities of monitoring the quality of care, especially for frail and disabled older adults living at home (Gulestø et al., 2025; Calkins, 1995), with the resulting risk of becoming “stuck in place” (Kvæl, 2025). Graybill and colleagues (2014) argue, “Focusing exclusively on monetary change as an outcome is problematic as it is possible for an intervention to have an “economic benefit,” but without improving the health and wellbeing of participants.” In an attempt to focus on aging in place from a more holistic perspective, they emphasize the need to consider not only economic factors but also all aspects of health and quality of life for those who choose to age in place. Some scholars highlight the importance of considering the economic status of governments, particularly in the era of austerity, when cuts to social spending have eroded key infrastructures for older adults (Van Hoof et al., 2021). This has shifted care responsibilities to families and volunteers, often leading to burnout and masking neoliberal agendas of privatizing the costs of aging. As a result, the aspiration to age in place can become unrealistic, especially for vulnerable groups and in contexts lacking adequate public services and transport (Drilling et al., 2025; OECD, 2025).

The definition of aging in place remains an open question. In a literal sense, it refers to aging in the place where one has always lived, but it is a widely used term with different definitions, including functional, symbolic, and emotional attachment to and the importance of homes, neighborhoods, and communities (Wiles et al., 2012). Several disciplines look at aging in place from different perspectives, and its definition has evolved over time. As Rogers et al. (2020) suggest, there is huge variability in the use of the term in different contexts. To better understand this subject, we believe that it is important to better define the term, with the aim of arriving at a comprehensive definition capable of integrating the heterogeneity and complexity of the construct. The theme of aging in place prompts significant reflection across various fields, raising important questions about the ambiguity of its definition.

The complex scenario underscores the need for greater attention to the aging population, particularly regarding the psychological characteristics that this age brings (Morganti, 2022). It highlights the urgent need to move beyond the stereotype of the old age as vulnerable, dysfunctional, unproductive, and excessive consumers of collective resources, often codified as “risk” (Morganti, 2024; Buffel and Phillipson, 2024; Talarsky, 1998). This shift is essential to promote future initiatives that support the rights and responsibility (empowerment) of individuals to age in the context in which they live.

In trying to summarize the different definitions assigned to aging in place, we keep in mind three main categories, as posited by Rogers et al. (2020): people, places, and time. Additionally, we introduce a new perspective and way of thinking about what lies in the thin space between individuals and places and the interplay between the two. We believe that exploring this interaction can enhance our understanding of the responsibility for healthy longevity and could be a crucial factor in aging in place studies.

### Aging in place for independent living

As mentioned above, from an individual perspective, some scholars describe aging in place as “remaining living in the community, with some level of independence, rather than in residential care” (Davey et al., 2004), emphasizing the importance of maintaining a degree of independence as opposed to aging in an institutional setting or an assisted living environment. Another example is the definition given by Horner and Boldy (2008), who describe aging in place as a “positive approach to meeting the needs of the older person, supporting them to live independently or with some assistance for as long as is possible”.

We believe that supporting the idea of aging in place as an agent of independence has a dual value. On the one hand, it could help the welfare economy of a country, as argued by Pynoos et al. (2008); on the other hand, it encourages the old people, or people who are about to grow old, to take responsibility for their own aging process from an active aging perspective. We believe that support for aging in place must reflect a new paradigm shift away from the stereotypical and ageist vision of old people toward a vision of empowered aging to sustain healthy longevity.

As the study of aging has been influenced by the medical sciences, aging has been viewed as an “innate and immutable process” (Powell and Hendricks, 2009). Therefore, the aging population has been seen as a “problem” and/or “economic burden” for society (Yi et al., 2016; Bai et al., 2016; Phillipson, 2013). Social theories of aging have bridged the gap between the biological and social aspects of aging, allowing for a comprehensive view of old age within the context of the entire life course (Hasworth and Cannon, 2015). However, the economic, environmental, and social paradigm in which the issue is currently framed still seems far from a bivariate vision of health and instead adheres to a medicalized vision of old age. The major challenge of our time is to shift toward a vision of old age that is far removed from ageist stereotypes (Butler, 1975), which often depict it as a time of loss. It is time to think about a culture of aging that embraces all stages of life, one that perceives old age not as a reality to be excluded from one’s life plan but as an integral part of life itself (Morganti, 2022). It is also important to emphasize the active role that older people can play in their own life trajectory to maintain their self-determination and empowerment for healthy longevity. To pursue this goal, the WHO adopted an initiative to combat ageism in 2016 (World Health Organization, 2021), and more recently, a “carta against ageism in healthcare” was agreed upon by scholars worldwide (Ungar et al., 2024).

### Aging in place and relationships

The WHO’s vision of aging in place entails allowing people “to remain at home in their familiar surroundings and maintain the relationships that are important to them” (World Health Organization, 2020), emphasizing the importance of connection and relationships. The social dimension is also pointed out in the WHO’s framework for age-friendly cities and communities (World Health Organization, 2007), which, as we have seen above, underpins the drive to age in the “right” place. However, from a psychological point of view, the relational dimension is crucial. The experience of living in a familiar environment may not be sufficient if it compromises individuals’ ability to successfully engage in activities of daily living. This perspective may be overly restrictive, potentially resulting in negative connotations and limiting opportunities for older adults (Weil and Smith, 2016). Many studies agree that social behavior plays an important role in supporting both psychological and physical health across the lifespan (Rothwell et al., 2023). There is evidence that social networks decline around the age of 60, and people tend to invest more energy in fewer relationships, with a particular emphasis on rewarding, positive interactions (English and Carstensen, 2014). High-quality social relationships seem to be associated with wellbeing across the lifespan, to the point that some researchers suggest that they may have an impact on longevity (Sirén et al., 2023; Holt-Lunstad et al., 2010; House et al., 1988). In general, social integration seems to be a protective factor against the frailty trajectory of aging, favoring longevity, whereas its absence has a detrimental effect. A meta-analysis conducted by Holt-Lunstad et al. (2015) found that social isolation was the best predictor of death under the age of 65, supporting another study conducted by Hawkley and Cacioppo (2007).

From a neuropsychological point of view, social engagement appears to be protective against the frailty trajectory of aging. Indeed, it plays an important role in cognitive function and successful aging (Bourassa et al., 2017). Several studies have shown a correlation between social activities and cognition, especially in aging adults (Bourassa et al., 2017). At the same time, perceived social isolation seems to be correlated with a major risk for cognitive decline (Cacioppo and Hawkley, 2009), although other studies have provided mixed evidence for the prediction of cognitive decline by social activity.

The stress hypothesis suggests that people with higher levels of social activity have lower levels of psychological stress, which could impair cognitive functioning (Bourassa et al., 2017). Additionally, research indicates a connection between reduced social integration and a higher risk of depression, with some studies showing that less social integration is associated with increased psychological distress, such as depression (e.g., Glass et al., 2006). Bourassa et al. (2017) provide further evidence that social participation is associated with memory and executive function over time in aging adults. The results of this study imply that interventions aimed at enhancing cognition by promoting social engagement are promising and may prove to be as effective, if not more so, than interventions targeting other factors associated with cognitive decline, such as depression, health status, and physical activity levels. This is also confirmed by a recent study, which highlights that maintaining a perceived good quality of life, together with a good level of cognitive reserve and autonomy in daily activities, represents a fundamental prerequisite for healthy aging, reducing the likelihood of developing depressive disorders (Gattuso et al., 2024).

### Aging in place in space and time

The study and planning of spaces to support aging in place are becoming increasingly important. Several studies highlight how the morphological and functional organization of neighborhoods, as well as the role of communities, are fundamental determinants of healthy longevity (Oswald et al., 2011). In this regard, space has become a central element in research on aging. The concept of “place” goes far beyond the physical dwelling, encompassing the neighborhood and the wider community, as from a psychological viewpoint, it contributes to the construction of an individual’s sense of identity. In this sense, it is therefore essential to distinguish between the two interpretations of this concept: one focusing on physical and functional aspects, and the other describing place in more psychological and experiential terms (Pani-Harreman et al., 2021). “Place attachment” (Rowles, 1983) plays a key role in this context, as it encompasses emotional bonds, familiarity, social connections, a sense of security, and the identity that individuals develop with their living environment, which tends to strengthen over time (Lebrusán and Gómez, 2022; Coleman et al., 2016; Hillcoat -Hillcoat-Nallétamby and Ogg, 2014; Wiles et al., 2012).

In the debate over the definition of aging in place, some scholars argue that certain aspects of the construct need to be better explored. Ahn et al. (2020), for example, suggest that the conceptual foundation of the idea of aging in place has not been sufficiently explored. Meanwhile, Lebrusán & Gómez (2022) suggest that the mechanisms through which place attachment develops need to be explored in depth, despite the evidence of strong associations between a number of place attachment dimensions and aging in place preferences (Clark et al., 2024).

Yarker et al. (2024), on the other hand, underline the necessity to better define the place as a concept itself, addressing the challenge highlighted by Andrews and colleagues (2013), as cited in Yarker et al. (2024). For this reason, underscoring the aspect of inequality of places, they emphasize the need to better comprehend the two aspects of places—territorial and relational—while recognizing the interdependence between the two. The debate, in fact, should not be reduced solely to the level of individual frailty but rather understood within the broader context of what the environment can provide in terms of opportunities and resources. The ability to remain at home, for instance, depends not only on individual health status and/or socio-economical personal possibilities but also on the availability of adequate housing conditions, community services, and urban resources that enable older adults to compensate for emerging difficulties, as described in the SOC- Selection, Optimization, and Compensation - model for aging (Baltes, 1990). From this perspective, aging in place cannot be considered an inherently optimal solution for everyone, since its feasibility and desirability are strongly mediated by contextual and structural factors.

In general, in the definition of aging in place, the dimension of place takes on the relevance and profound meaning of the heterogeneous corpus of reflections that it entails. As suggested by Yarker et al. (2024), it is vital to take into account the notion of non-neutral space, which encompasses various meanings, including symbolic and identity-related aspects.

In addition, Rogers et al. (2020) suggest that aging in place should be defined as “one’s journey to maintain independence in one’s place of residence as well as to participate in one’s community”, underlying the dimension of aging in place as a process and supporting the idea that it is not limited to one’s physical place, which in turn plays a fundamental role, as it is itself subject to change over time (Lewis and Buffel, 2020).

All the perspectives and suggestions outlined above imply going beyond the single categories of this concept and paying attention to their intersectionality. The definition of aging in place proposed by Rogers et al. (2020) aligns precisely with this discourse, viewing time as a process that spans the entire lifespan. The term “journey” used by Rogers and colleagues (2020) denotes the change people’s experience over time in the aging process. Moreover, this definition is congruent with psychological research on aging considered from a lifespan perspective (Morganti, 2022) and with aging considered as a kind of “new map of life” (Stanford Center on Longevity, 2024), which calls for new initiatives to promote longevity.

It is key to look at aging as a trajectory along which changes will occur and how individuals can put into practice compensatory skills aimed at maintaining their state of flow and wellbeing. For this reason, we have decided to opt for the last definition of aging in place, which we consider the most complete. In addition, we propose reflecting on something that has been less considered: the thin space between individuals and their contexts as an opportunity for action to promote healthy longevity.

## The interplay between individuals and contexts as affordance for healthy longevity

Starting from a situated and embodied cognition approach (Morganti, 2020), we suggest that there may be a dimension between people, places, and individuals that could benefit from further exploration, particularly in the context of the different dimensions of aging in place, with a particular focus on research that incorporates an individual and collective psychological perspective. Accordingly, space can be seen as closely linked to an individual’s possibilities for action while also being shaped by the characteristics of the same individual exploring that space. We are referring to something that, according to *The Ecological Approach to Visual Perception* (Gibson, 1977), “is neither an objective property nor a subjective property; or is both if you like.” In defining this continuous clamping between organisms and environments, Gibson introduces a new term: *affordance*. The concept of *affordance* emphasizes the potential actions or opportunities for interaction that the environment offers to each organism and that the organism can play out in the environment according to its neural architecture and body structure. This emphasizes the importance of studying not only the characteristics of individuals (such as the aging person, who is our focus of interest) and places (which, as mentioned, is the urban environment) but also the relationship between them.

Affordance is not just a passive process of receiving sensory information from the environment; it is also an active process of perceiving opportunities for action within that environment. Thus, the term *affordance* refers “to both the environment and the animal in a way that no existing term does. It implies the complementarity of the animal and the environment” (Gibson, 1977). In this regard, it is essential to distinguish between natural affordances and conventional affordances (Ramstead et al., 2016). Natural affordances are action possibilities whose use depends on an organism’s ability to exploit reliable correlations in the environment; for example, given a human’s ability to walk, an unpaved road offers the possibility of a walk. Conventional affordances, on the other hand, are action possibilities that depend on agents’ ability to leverage explicit or implicit expectations, norms, conventions, and cooperative social practices. This means that, in the context of older adults, not only the living space but also interacting with others and understanding their modes of thought and action are crucial for the emergence of social and cultural opportunities.

For this reason, the contexts in which people are living must be studied in relation to the specific characteristics of the individuals who use them. Accordingly, place characteristics are not simply neutral properties of the environment and cannot be measured in terms of purely spatial dimensions. Each time individuals are “in place” (as we would like to define the aging trajectory), affordances are inherent properties of the environment that are directly perceived by organisms based on their bodily capabilities and activity intentions (e.g., a chair affords sitting if the agent intends to use it to get some rest and if, at the same time, its body allows it to switch from a standing to a sitting position). This also implies that affordances are often produced relationally through a combination of natural and conventional affordances (Söderström et al., 2025). As the characteristics of a place change, or the type of organism in that place changes, so do the affordances of that peculiar spatial perception. To better understand this, it is useful to distinguish between the landscape of affordances, which is the set of all affordances available in an environment, and the field of affordances, which corresponds to the affordances with which an organism actually engages (Ramstead et al., 2016). This distinction between a set of possibilities and those that are activated is crucial. Affordances do not appear as discrete elements, but as a “matrix of affordances with its own structure or configuration”. For example, a preferred bench overlooking the valley becomes a resource in the field of affordances if it is accessible along a suitable pedestrian path.

This is why we believe that in studying aging in place, it is not possible to analyze urban spaces without closely examining individuals and place characteristics in a life-course perspective in order to understand whether the interplay between them represents an opportunity or a limitation as in the affordance theory.

Next, it is important to underscore that the implication of the concept of affordance is rooted not only in the relationship between people and places but also among individuals. Gibson (1977) says that “the richest and most elaborate affordances of the environment are provided by other animals and, for us, other people”. People move in space, and their movements are animated. In this way, “behavior affords behavior”. All observable behaviors in a social context depend on the perception—or sometimes the misperception—of what other individuals afford. In other words, we can say that in every system, there is “mutual affordance” that we must consider. This can mean that not only objects in the world (e.g., physical features of the environment, amenities, obstacles, etc.) can provide affordances for the individual but also that while living in a social environment, every gesture, act of communication, or emotion expressed by another may constitute an affordance for an interpersonal relationship. In this context, conventional affordances are particularly relevant, as social interactions and the understanding of mutual expectations are fundamental (Söderström et al., 2025).

In sum, from a situated cognition approach, we propose the affordance as a point of intersection between people and places, as a concept that we would like to put in the preposition “*in*” that connects *aging* and *place*, analyzing place as a set of opportunities that emerges in the continuous and reciprocal interplay between the individual and the environment. It is in this *thin space* that we can grasp the opportunity for healthy longevity. For this reason, we believe that we should add to the definition of Rogers et al. (2020) the concept of affordable spaces, defining space as not something neutral but as a continuous opportunity for individuals to act. It is our contention that only from the analysis of urban spaces, meant as limits or possibilities in everyday life, can we grasp how the city (and its structural and relational elements) can become an adequate place to empower individuals to age healthily.

## A situated psychological perspective for aging in place studies

In light of what has been discussed so far, we believe that studies on aging in place should consider more deeply the interplay between people and places. To this end, a complex epistemological approach may be beneficial in capturing the heterogeneity of both aging individuals and the places where their lives occur. This could help us seize opportunities to support individual choices for living and healthy longevity. Indeed, as discussed above, in reviewing the literature on aging in place, a definition of an evolving (and thus aging) individual seems to be lacking, thus failing to account for the complexity of the human being, which reaches its highest expression in aging (Morganti, 2024). For this reason, it is necessary to adopt an epistemology of complexity for the study of the multiple possibilities that an aging person may have when exploring and using the urban space in which they live. When considering the many facets that comprise the everyday life of an older adult (unless we are willing to consider said adult solely and exclusively as an undistinguished individual unable to enjoy their surrounding space, a position that we frankly do not feel able to support!), it is important to recognize that each individual has unique characteristics and experiences within their environment. While it is valuable to analyze individuals and their surroundings, it is also essential to recognize the diverse roles that individuals may assume in their daily lives. However, it should be noted that the cultural context in which research is embedded is always crucial for the interpretation and application of findings.

This perspective aims to encourage the idea of the empowerment of the aging individual, dismissing stereotypes that tend to homogenize older adults and label them as frail and a social burden. Furthermore, it could also be a way to involve them in research and to enable them to be empowered towards their own aging trajectory.

**FIGURE 1:**
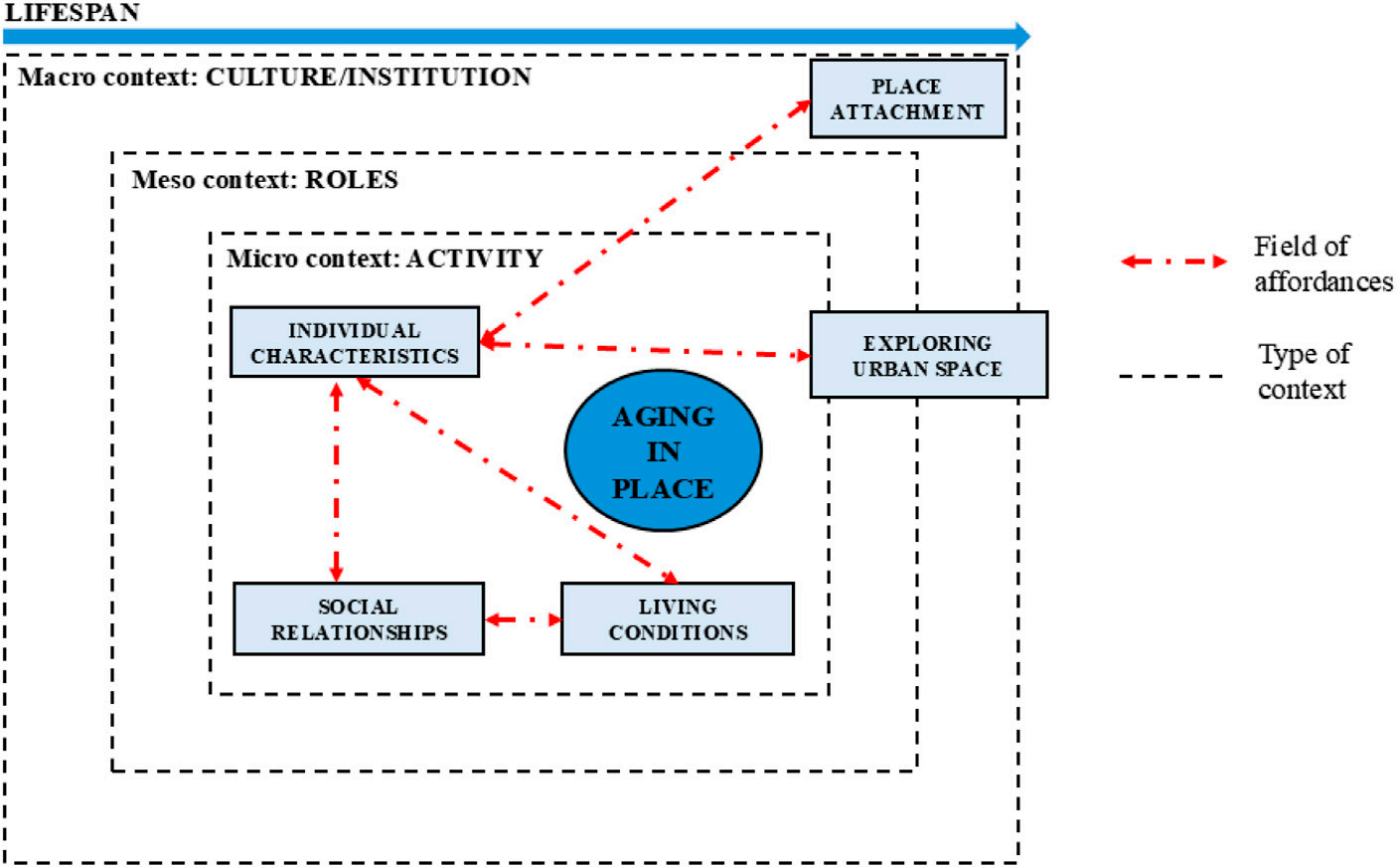
Ecological development for aging in place conceptual framework during lifespan driven by affordances.

In light of the above, and in accordance with Riekkola et al. (2024), we suggest that Bronfenbrenner (1979) could be a valuable addition to the current approach. Bronfenbrenner describes human development as the result of interactions between the individual and different levels of the surrounding environment, organized into a series of reciprocally linked systems. This model places equal importance on individuals’ aging process, place choices, and creation of proximal relationships, as well as the role of the entire system that surrounds and influences them. In line with the Bronfenbrennerian vision, we consider it is necessary to observe people and contexts from different perspectives and on multiple levels. This could begin by observing the individual who stands at the micro-level context that constitute the center of the entire system of activity and human development. Observation has also to start from the micro-level’s surrounding contexts and consider the individuals interacting within meso-level contexts (e.g., the different possible roles that individuals can take on in order to participate in the action). At the same time observation has to consider the institutional constraints and the influence of the cultural characteristics of the macro-level context when the individual is engaged in an interaction. As in every other moment of human life and evolution, it is impossible to ignore the analysis of each of these systems (which are strongly intertwined) when analyzing human development from birth to old age. In sum, this ecological evolution must not overlook the place where it occurs and the affordances that the different contextual levels observed can offer to the individual. Accordingly, in this article, we propose a psychological perspective for studying aging in place that draws on Bronfenbrenner’s ecological theory and the concept of affordances as complementary and indispensable frameworks. The individual must be conceptualized beyond the confines of a spatial explorer or an evolving organism situated solely within a microsystem (therefore in direct interaction with a single sphere of activity) if our objective is to define optimal aging in place solutions for the individual. Instead, in this dynamic interaction between individual and environment provided by affordances, it is imperative to consider the comprehensive array of systems–meso, eso and macrosystems–delineated in Bronfenbrenner’s ecological model. From this standpoint, an examination of “thin space” becomes congruent with the concept of ecological development and situated cognition, a peculiar perspective in psychology that consider the affordances as the medium of space knowledge and utilization. In Figure 1 an explanation of how affordances drive the ecological development for aging in place during lifespan.

Our integrated framework constitutes the innovative contribution that is intended to be made to the study of aging in place, and it clearly differentiates the present theoretical position from the approaches that have already been extensively developed within the field of urban studies and geographical gerontology.

As mentioned above, consistent with the Bronfenbrennerian vision, we consider it necessary to observe people and contexts by adopting different lenses on multiple levels, starting from the individual level, which corresponds to the micro-level context of the entire system. The analysis of the individual should be conducted both from a quantitative point of view in order to obtain a health status assessment of the subject, and from a qualitative point of view, to comprehend the aging individual’s life history, their motivation for choosing to live in the city, and their perceived satisfaction when interacting with their chosen urban environment, while also considering their socio-economic conditions. We believe that qualitative research can significantly enhance our understanding of visible aspects, but only if the researcher is embedded within a culturally determined system. Furthermore, it can involve older people in the research process, empowering them to shape their own aging trajectories.

In sum, in order to facilitate research that can establish a comprehensive understanding of the process of aging from a lifespan perspective, it is imperative to strike the right balance between qualitative and quantitative methodological choices. For this reason, we advise the use of mixed methods analysis (Curry et al., 2009; Phillips et al., 2013).

At last, it is our intention to advocate for a perspective that observes people in their contexts. Given that we hold the view that it is equally important to grasp the potential of the environment for positive action in order to ascertain whether it may act as a hindrance or opportunity for positive aging outcomes, we suggest that an in-depth analysis of urban spaces be conducted. This analysis should consider various elements, including demographic composition, historical narratives of the places, and their spatial organization. Furthermore, we think it would be valuable to analyze the mediating role of the contexts in which each individual is acting to better understand the role that each agent assumes in such contextualized action.

For this reason, we suggest combining spatial analysis with ethnographic participant observation, which could observe places, people, and their relationships from different points of view (Abramson, 2021; Geertz and Leonini, 1988). To this end, “go-along” interviews could represent a particularly effective tool (Söderström et al., 2025). This method involves accompanying participants during their daily or habitual walks, allowing researchers to record conversations and take photographs of significant places, obstacles, or natural/conventional affordances.

Ethnographic research that combines participatory geospatial and qualitative methods is emerging in the field of aging and promises to explore the complex person–place relationships (Aw et al., 2021; Hand et al., 2017). However, it is the contention of the present argument that for a comprehensive ethnographic approach to observing older individuals in urban environments, it is essential to extend the analysis beyond merely describing *how* they move or interact physically within the city. In fact, more to where they go in an urban place, it is imperative to include, for a more thorough understanding, the roles and deeper motivations they assume when interacting with space. This is crucial because, as in Bronfenbrenner’s perspective, the mere act of movement (the “how”) is often the external manifestation of complex internal and relational factors (the “why” and the “who”), which profoundly influence the experience of aging and the construction of community. The motivations for going out and moving around are driving forces that push older adults to interact with the urban environment, going far beyond mere physical ability and can be interpreted as in the SOC model. Some studies, for example, highlight that social engagement and the preservation of identity are such strong motivations for community mobility that they can “override” health problems, pain, functional limitations, and hazardous conditions (Gardner, 2014; Krogstad et al., 2015). Thus, a comprehensive approach requires immersing oneself in the experiences of older adults, starting from their specific physical and psychological characteristics and extending to an understanding of the cultural, social, and personal meanings they attribute to their movements and interactions, while recognizing how these intrinsic factors shape not only their behavior but also the very nature of the urban spaces in which they live and age. It is important to understand not only the action possibilities present in the city (e.g., architectural barriers and pedestrian facilities), but also which of these are presenting affordances for them and are consequently chosen in using home and urban space. For this reason, we refer to a “thin space,” which is composed not only of older adults’ physical and psychological characteristics—an important element often overlooked in studies on urban aging—but also of their intentions, emotions and social roles while living in places. In accordance with the principles of affordances, it is evident that these locations do not remain static; rather, they possess the capacity to influence and be influenced by the individuals who traverse them, reside in them, and cultivate relationships within their confines.

As demonstrated by Torres (2025), this offers an in-depth perspective on the challenges and adaptation strategies of older adults in metropolitan areas. This two-pronged approach will provide a multidimensional and multi-level picture of space affordances and will highlight the elements to consider, improve, or integrate within the city to offer real opportunities to enhance individuals’ healthy aging. Without an in-depth analysis of people, places, and their relationships, there is a risk of delivering a blurred representation of the observation subject. As Van Hoof et al. (2021) underscore, many planning principles are taken as universal facts; however, they reveal an underdeveloped sense of our aging society. This reflection led him to conclude that it is crucial to reflect on the gap between what we think we know about old people and who they really are and what they really need.

As previously stated, it is our conviction that the analysis should be conducted using a mixed methods approach comprised of both quantitative and qualitative approaches. Quantitative analysis would allow us to gain an understanding of the health status of the individuals and the characteristics of the environment, while qualitative analysis would help us obtain insights into the aging individual’s life and place history, their motivation behind individuals’ living choices in the city, and their perceived satisfaction with the interactions in the urban environment they have decided to live in. Qualitative methods can provide valuable insights that supplement the findings from quantitative research. In our view, to gain a comprehensive understanding of the aging process across the lifespan, it would be valuable to strike a balance between qualitative and quantitative methodologies.

Our view is that by adopting this approach to research through a multidimensional and multi-level methodology, it will become possible to consider and create environments that support the idea of an empowered aging process, leading to healthier, longer lives.

In conclusion, it is becoming increasingly clear that there is no single universal experience of aging. Rather, the aging process is shaped by a multitude of factors, including urban and cultural differences. This represents a significant shift in perspective in the study of aging. While the topic has been widely studied in other parts of the world, there is still much to be done to adapt it to the Italian culture of living. It would be beneficial to have a vision that takes a stand regarding the space that exists between people and places, which is strictly dependent on the culture of the people and the meanings of the places. If we examine the few existing Italian studies on aging in place, we see that they are mostly from the domains of the economic sciences, architecture, or urban planning. It is therefore important to consider that if these studies do not consider older people as individuals who implement choices of action in relation to the affordances that the city in which they are located offers them, they may risk being short-sighted. In view of the paucity of literature on this subject, it is important to consider the unique experiences of Italian organizations and the concept of aging in place. The recognition of diversity among older people’s aging experiences and the understanding that these experiences can be shaped and influenced by urban and cultural differences represent a fundamental perspective shift in the study of aging. We believe that by adopting a research model characterized by a multidimensional and multi-level approach, it will be possible to design physical and social spaces that support empowered aging and promote healthy longevity.

## Data Availability

The original contributions presented in the study are included in the article/supplementary material, further inquiries can be directed to the corresponding author.

## References

[B1] Abdullah B. Wolbring G. (2013). Analysis of newspaper coverage of active aging through the lens of the 2002 world health organization active ageing report: a policy framework and the 2010 Toronto charter for physical activity: a global call for action. Int. J. Environ. Res. Public Health 10 (12), 6799–6819. 10.3390/ijerph10126799 24317386 PMC3881142

[B2] Abramson C. M. (2021). “Ethnographic methods for research on aging: making use of a fundamental toolkit for understanding everyday life,” in Handbook of aging and the social sciences (Academic Press), 15–31.

[B3] Ahn M. Kwon H. J. Kang J. (2020). Supporting aging-in-place well: findings from a cluster analysis of the reasons for aging-in-place and perceptions of well-being. J. Appl. Gerontology 39 (1), 3–15. 10.1177/0733464817748779 29277156

[B4] Andrews G. J. Evans J. Wiles J. L. (2013). Re-spacing and re-placing gerontology: relationality and affect. Ageing and Soc. 33 (8), 1339–1373. 10.1017/s0144686x12000621

[B5] Aw S. Koh G. C. Oh Y. J. Wong M. L. Vrijhoef H. J. Harding S. C. (2021). Interacting with place and mapping community needs to context: comparing and triangulating multiple geospatial-qualitative methods using the focus–expand–compare approach. Methodol. Innov. 14 (1), 2059799120987772. 10.1177/2059799120987772

[B6] Bai X. Lai D. W. Guo A. (2016). Ageism and depression: perceptions of older people as a burden in China. J. Soc. Issues 72 (1), 26–46. 10.1111/josi.12154

[B7] Baltes P. B. (1990). “Psychological perspectives on successful aging: the model of selective optimization with compensation,” in Successful aging: perspectives from the behavioral sciences/the european science foundation, university of cambrige, Cambridge.

[B8] Beard J. R. Petitot C. (2010). Ageing and urbanization: can cities be designed to foster active ageing? Public Health Rev. 32, 427–450. 10.1007/bf03391610

[B9] Bigonnesse C. Chaudhury H. (2020). The landscape of “aging in place” in gerontology literature: emergence, theoretical perspectives, and influencing factors. J. Aging Environ. 34 (3), 233–251. 10.1080/02763893.2019.1638875

[B10] Bigonnesse C. Chaudhury H. (2022). Ageing in place processes in the neighbourhood environment: a proposed conceptual framework from a capability approach. Eur. J. Ageing 19, 63–74. 10.1007/s10433-020-00599-y 35250420 PMC8881541

[B14] Boudiny K. (2013). Active ageing’: from empty rhetoric to effective policy tool. Ageing and Soc. 33 (6), 1077–1098. 10.1017/S0144686X1200030X 23913994 PMC3728916

[B15] Bourassa K. J. Memel M. Woolverton C. Sbarra D. A. (2017). Social participation predicts cognitive functioning in aging adults over time: comparisons with physical health, depression, and physical activity. Aging and Ment. Health 21 (2), 133–146. 10.1080/13607863.2015.1081152 26327492

[B16] Bowling A. Dieppe P. (2005). What is successful ageing and who should define it? Br. Med. J. 331 (7531), 1548–1551. 10.1136/bmj.331.7531.1548 16373748 PMC1322264

[B17] Bronfenbrenner U. (1979). The ecology of human development: experiments by nature and design. Harvard University Press.

[B18] Buffel T. Phillipson C. (2024). Ageing in place in urban environments. London: Routledge. 10.1007/s10433-025-00875-9 PMC12440835 40956514

[B19] Buffel T. Handler S. Phillipson C. (2018). “Age-friendly cities and communities,” in A global perspective. Bristol. Policy. (Ageing in a Global Context). Bristol: Policy Press.

[B20] Butler R. N. (1975). Why survive? Being old in America. Harper & Row.

[B22] Cacioppo J. T. Hawkley L. C. (2009). Perceived social isolation and cognition. Trends Cognitive Sci. 13 (10), 447–454. 10.1016/j.tics.2009.06.005 19726219 PMC2752489

[B23] Calkins M. P. (1995). From aging in place to aging in institutions: exploring advances in environments for aging. Gerontologist 35, 567–571. 10.1093/geront/35.4.567

[B24] Clark D. (2000). World urban development: processes and patterns at the end of the twentieth century. Geogr. J. Geogr. Assoc. 85 (1), 15–23. 10.1080/20436564.2000.12219727

[B25] Clark W. A. Ong Viforj R. Phelps C. (2024). Place attachment and aging in place: preferences and disruptions. Res. Aging 46 (3–4), 179–196. 10.1177/01640275231209683 37909287 PMC10868147

[B26] Coleman T. Kearns R. A. Wiles J. (2016). Older adults’ experiences of home maintenance issues and opportunities to maintain ageing in place. Hous. Stud. 31 (8), 964–983. 10.1080/02673037.2016.1164834

[B27] Curry L. A. Nembhard I. M. Bradley E. H. (2009). Qualitative and mixed methods provide unique contributions to outcomes research. Circulation 119 (10), 1442–1452. 10.1161/CIRCULATIONAHA.107.742775 19289649

[B28] Davey J. A. de Joux V. Nana G. Arcus M. (2004). Accommodation options for older people in aotearoa/new zealand. Research: Centre for Housing, 1–204.

[B29] Drilling M. Suero P. Al-Shoubaki H. Neuhaus F. (2025). Ageing and urban planning. London: Taylor and Francis, 362.

[B30] English T. Carstensen L. L. (2014). Selective narrowing of social networks across adulthood is associated with improved emotional experience in daily life. Int. J. Behav. Dev. 38 (2), 195–202. 10.1177/0165025413515404 24910483 PMC4045107

[B31] Foster L. Walker A. (2015). Active and successful aging: a European policy perspective. Gerontologist 55 (1), 83–90. 10.1093/geront/gnu028 24846882 PMC4986585

[B32] Foster L. Walker A. (2021). Active ageing across the life course: towards a comprehensive approach to prevention. BioMed Res. Int. 2021 (1), 6650414. 10.1155/2021/6650414 33623785 PMC7875625

[B33] Gardner P. (2014). The role of social engagement and identity in community mobility among older adults aging in place. Disabil. Rehabilitation 36 (15), 1249–1257. 10.3109/09638288.2013.837970 24099580

[B34] Geertz C. Leonini L. (1988). Antropologia interpretativa. Bologna: Il Mulino, 192.

[B35] Gibson J. J. (1977). “The theory of affordances,” in perceiving, acting, and knowing: toward an ecological psychology. Editors Shaw R. Bransford J. (Hillsdale, NJ: Erlbau), 67–82.

[B36] Glass T. A. De Leon C. F. M. Bassuk S. S. Berkman L. F. (2006). Social engagement and depressive symptoms in late life longitudinal findings. J. Aging Health 18 (4), 604–628. 10.1177/0898264306291017 16835392

[B37] Golant S. M. (2020). The distance to death perceptions of older adults explain why they age in place: a theoretical examination. J. Aging Stud. 54, 100863. 10.1016/j.jaging.2020.100863 32972627 PMC7489887

[B38] Graybill E. M. McMeekin P. Wildman J. (2014). Can aging in place be cost effective? A systematic review. PLoS One 9 (7), e102705. 10.1371/journal.pone.0102705 25058505 PMC4109953

[B39] Greenberg B. R. Schwarz J. (2012). Aging in place … with a little help from our friends: an overview for grantmakers about aging in the community. New York: The Philantropic group.

[B40] Gulestø R. J. A. Ågotnes G. Glasdam S. (2025). Ageing in place’in Norway–A Fairclough-inspired discourse analysis of a white paper. Health and Place 94, 103497. 10.1016/j.healthplace.2025.103497 40479872

[B41] Hammarström G. Torres S. (2012). Variations in subjective well-being when ‘aging in place’—A matter of acceptance, predictability and control. J. Aging Stud. 26, 192–203. 10.1016/j.jaging.2011.12.004

[B42] Hand C. Huot S. Laliberte Rudman D. Wijekoon S. (2017). Qualitative–geospatial methods of exploring person–place transactions in aging adults: a scoping review. Gerontologist 57 (3), e47–e61. 10.1093/geront/gnw130 28069885

[B43] Hasworth S. B. Cannon M. L. (2015). Social theories of aging: a review. Disease-a-Month DM 61 (11), 475–479. 10.1016/j.disamonth.2015.09.003 26519983

[B44] Hawkley L. C. Cacioppo J. T. (2007). Aging and loneliness: downhill quickly? Curr. Dir. Psychol. Sci. 16 (4), 187–191. 10.1111/j.1467-8721.2007.00501.x

[B45] Hillcoat-Nallétamby S. Ogg J. I. M. (2014). Moving beyond ‘ageing in place’: older people's dislikes about their home and neighbourhood environments as a motive for wishing to move. Ageing and Soc. 34 (10), 1771–1796. 10.1017/s0144686x13000482

[B46] Holt-Lunstad J. Smith T. B. Layton J. B. (2010). Social relationships and mortality risk: a meta-analytic review. PLoS Med. 7 (7), e1000316. 10.1371/journal.pmed.1000316 20668659 PMC2910600

[B47] Holt-Lunstad J. Smith T. B. Baker M. Harris T. Stephenson D. (2015). Loneliness and social isolation as risk factors for mortality: a meta-analytic review. Perspect. Psychol. Sci. 10 (2), 227–237. 10.1177/1745691614568352 25910392

[B48] Horner B. Boldy D. P. (2008). The benefit and burden of ageing-in-place in an aged care community. Aust. Health Rev. 32, 356–365. 10.1071/ah080356 18447827

[B49] House J. S. Landis K. R. Umberson D. (1988). Social relationships and health. Science 241 (4865), 540–545. 10.1126/science.3399889 3399889

[B50] Krogstad J. R. Hjorthol R. Tennøy A. (2015). Improving walking conditions for older adults. A three-step method investigation. Eur. J. Ageing 12 (3), 249–260. 10.1007/s10433-015-0340-5 28804358 PMC5549237

[B51] Kvæl L. A. H. (2025). Ageing in place or stuck in place: a critical qualitative study on older adults’ independence across six municipalities in Norway. Soc. Sci. and Med. 375, 118098. 10.1016/j.socscimed.2025.118098 40267761

[B52] Lebrusán I. Gómez M. V. (2022). The importance of place attachment in the understanding of ageing in place: “the stones know me.”. Int. J. Environ. Res. Public Health 19 (24), 17052. 10.3390/ijerph192417052 36554931 PMC9779384

[B53] Lewis C. Buffel T. (2020). Aging in place and the places of aging: a longitudinal study. J. Aging Stud. 54, 100870. 10.1016/j.jaging.2020.100870 32972616

[B54] Lofqvist C. Granbom M. Himmelsbach I. Iwarsson S. Oswald F. Haak M. (2013). Voices on relocation and aging in place in very old age—A complex and ambivalent matter. Gerontologist 53, 919–927. 10.1093/geront/gnt034 23626372

[B55] McGrath C. Hand C. (2021). Engagement in the social context of the neighbourhood: a critical ethnographic study of older adults with age-related vision loss. Wellbeing, Space Soc. 2, 100041. 10.1016/j.wss.2021.100041

[B56] MetLife Mature Market Institute (2010). “Aging in place 2.0,” in Rethinking solutions to the home care challenge. Available online at: http://www.metlife.com/assets/cao/mmi/publications/studies/2010/mmi-aging-place.pdf.

[B57] MichelJ. P. (2020). Editorial: urbanization and ageing health outcomes. J. Nutr. Health and Aging 24 (5), 463–465. 10.1007/s12603-020-1360-1 32346681

[B11] Morganti F. (2020). “Ecologia degli sviluppi: corpo, opportunità d’azione e cognizione situata,” in La Formazione in Psicomotricità. Editor Zatti L. B. e A. (Trento: Erikson).

[B12] Morganti F. (2022). Psicologia dell’Invecchiamento e qualità della vita. Salute, fragilità, demenze, 1314. Carocci Roma.

[B100] Morganti F. (2024). Longevity as a responsibility: Constructing healthy aging by enacting within contexts over the entire lifespan. Geriatrics 9 (4), 93. 39051257 10.3390/geriatrics9040093 PMC11270264

[B58] OECD (2025). Cities for all ages, OECD urban studies. Paris: OECD Publishing. 10.1787/f0c8fefa-en

[B59] Oswald F. Jopp D. Rott C. Wahl H. W. (2011). Is aging in place a resource for or risk to life satisfaction? Gerontologist 51 (2), 238–250. 10.1093/geront/gnq096 21097552

[B60] Pani-Harreman K. E. Bours G. J. Zander I. Kempen G. I. van Duren J. M. (2021). Definitions, key themes and aspects of ‘ageing in place’: a scoping review. Ageing and Soc. 41 (9), 2026–2059. 10.1017/s0144686x20000094

[B61] Pfaller L. Schweda M. (2019). Excluded from the good life? An ethical approach to conceptions of active ageing. Soc. Incl. 7 (3), 44–53. 10.17645/si.v7i3.1918

[B62] Phillips J. Walford N. Hockey A. Foreman N. Lewis M. (2013). Older people and outdoor environments: pedestrian anxieties and barriers in the use of familiar and unfamiliar spaces. Geoforum 47, 113–124. 10.1016/j.geoforum.2013.04.002

[B63] Phillipson C. (2013). Ageing. 1st ed. John Wiley and Sons Ltd.

[B64] Powell J. L. Hendricks J. (2009). The sociological construction of ageing: lessons for theorising. Int. J. Sociol. Soc. Policy 29 (1/2), 84–94. 10.1108/01443330910934745

[B65] Pynoos J. Nishita C. Cicero C. Caraviello R. (2008). Aging in place, housing, and the law. Elder Law Journal, 16 (1), 77–105.

[B66] Ramstead M. J. Veissière S. P. Kirmayer L. J. (2016). Cultural affordances: scaffolding local worlds through shared intentionality and regimes of attention. Front. Psychol. 7, 1090. 10.3389/fpsyg.2016.01090 27507953 PMC4960915

[B67] Riekkola J. Isaksson G. Lilja M. Rutberg S. (2024). Possibilities and challenges for older couples to continue ageing in place. J. Aging Stud. 69, 101229. 10.1016/j.jaging.2024.101229 38834252

[B68] Rogers W. A. Ramadhani W. A. Harris M. T. (2020). Defining aging in place: the intersectionality of space, person, and time. Innovation Aging 4 (4), igaa036. 10.1093/geroni/igaa036 33173834 PMC7595274

[B69] Rothwell E. S. Carp S. B. Bliss-Moreau E. (2023). The importance of social behavior in nonhuman primate studies of aging: a mini-review. Neurosci. and Biobehav. Rev. 154, 105422. 10.1016/j.neubiorev.2023.105422 37806369 PMC10716830

[B70] Rowles G. (1983). Place and personal identity in old age: observations from appalachia. J. Environ. Psychol. 3 (4), 299–313. 10.1016/s0272-4944(83)80033-4

[B71] Siltanen S. Keskinen K. E. Lahti A. M. Rantanen T. von Bonsdorff M. (2024). Active aging in senior housing residents and community-dwelling older adults: a comparative study in Finland. J. Aging Health 36 (5-6), 299–307. 10.1177/08982643231186627 37376762

[B72] Sirén A. L. Seppänen M. von Bonsdorff M. B. (2023). Social participation considered as meaningful in old age− the perceptions of senior housing residents in Finland. Ageing Int. 48 (4), 1238–1258. 10.1007/s12126-023-09522-z 37359716 PMC9989560

[B73] Sixsmith J. Sixsmith A. Fänge A. M. Naumann D. Kucsera C. Tomsone S. (2014). Healthy ageing and home: the perspectives of very old people in five European countries. Soc. Sci. and Med. 106, 1–9. 10.1016/j.socscimed.2014.01.006 24524960

[B74] Smith A. E. (2009). Ageing in urban neighbourhoods: place attachment and social exclusion. Bristol: Policy Press.

[B75] Söderström O. Zittoun T. Gfeller F. Ruggeri A. Kloepper I. (2025). Designing landscapes of affordances for ageing in place. Geogr. Helvetica 80 (1), 67–79. 10.5194/gh-80-67-2025

[B76] Stanford Center on Longevity (2024). The new map of life. Available online at: https://longevity.stanford.edu/the-new-map-of-life-initiative (Accessed April 30, 2024).

[B77] Stones D. Gullifer J. (2016). ‘At home it’s just so much easier to be yourself’: older adults' perceptions of ageing in place–CORRIGENDUM. Ageing and Soc. 37 (1), 219. 10.1017/s0144686x16000854

[B78] Talarsky L. (1998). Defining aging and the aged: cultural and social constructions of elders in the US. Arizona Anthropologist 13, 101–107.

[B79] Tinker A. (1999). Ageing in place: what can we learn from each other? The sixth F. Oswald barnett oration. Melbourne: Ecumenical Housing Inc. and Copelen Child Family Services.

[B80] Torres S. (2025). At home in the city: growing old in urban America. Oakland: Univ of California Press.

[B81] UNFPA (2007). State of world population 2007: unleashing the potential of urban growth. New York: United Nations Population Fund.

[B82] Ungar A. Cherubini A. Fratiglioni L. de la Fuente-Núñez V. Fried L. P. Krasovitsky M. S. (2024). Carta of florence against ageism: no place for ageism in healthcare. Journals Gerontology, Ser. A Biol. Sci. Med. Sci. 79 (3), glad264. 10.1093/gerona/glad264 38419345 PMC10902610

[B83] United Nations (2019). World population prospects 2019: highlights. Available online at: https://population.un.org/wpp/Publications/Files/WPP2019_Highlights.pdf.

[B84] United Nations (2024). World population prospects 2024: highlights. Available online at: https://population.un.org/wpp/assets/Files/WPP2024_Summary-of-Results.pdf.

[B85] Van Dyk S. (2014). The appraisal of difference: critical gerontology and the active-ageing-paradigm. J. Aging Stud. 31, 93–103. 10.1016/j.jaging.2014.08.008 25456626

[B86] Van Hoof J. Marston H. R. Kazak J. K. Buffel T. (2021). Ten questions concerning age-friendly cities and communities and the built environment. Build. Environ. 199, 107922. 10.1016/j.buildenv.2021.107922

[B87] Vasunilashorn S. Steinman B. A. Liebig P. S. Pynoos J. (2012). Aging in place: evolution of a research topic whose time has come. J. Aging Res. 2012 (1), 120952. 10.1155/2012/120952 22175020 PMC3227373

[B88] Weil J. Smith E. (2016). Revaluating aging in place: from traditional definitions to the continuum of care. Work. Older People 20 (4), 223–230. 10.1108/wwop-08-2016-0020

[B89] Wiles J. L. Leibing A. Guberman N. Reeve J. Allen R. E. (2012). The meaning of “aging in place” to older people. Gerontologist 52 (3), 357–366. 10.1093/geront/gnr098 21983126

[B90] World Health Organization. (2002). Active ageing: a policy framework. Geneve: World Health Organization. Available online at: https://iris.who.int/handle/10665/67215

[B91] World Health Organization. (2007). Global age-friendly cities: a guide. Geneve: World Health Organization. Available online at: https://iris.who.int/handle/10665/43755

[B92] World Health Organization. (2017). Global strategy and action plan on ageing and health. Geneve: World Health Organization. Available online at: https://iris.who.int/handle/10665/329960.

[B93] World Health Organization (2020). Global database of age-friendly practices. Geneve: World Health Organization. Available online at: https://extranet.who.int/agefriendlyworld/afp/.

[B94] World Health Organization. (2021). Global report on ageism. Geneve: World Health Organization. Available online at: https://iris.who.int/handle/10665/340208

[B95] World Health Organization. (2023). WHO clinical consortium on healthy ageing 2022: report of consortium meeting, 5–6 December 2022. Geneve: World Health Organization. Available online at: https://iris.who.int/handle/10665/372136.

[B96] World Health Organization (2025). Age-friendly world. About the global network for age-friendly cities and communities. Available online at: https://extranet.who.int/agefriendlyworld/who-network/ (Accessed September 10, 2025).

[B97] Yarker S. Doran P. Buffel T. (2024). Theorizing “place” in aging in place: the need for territorial and relational perspectives. Gerontologist 64 (2), gnad002. 10.1093/geront/gnad002 36655690 PMC10809216

[B98] Yi J. Lu D. Deng Y. (2016). The future of social elderly care in China: from the perspective of service-oriented government. J. Serv. Sci. Manag. 9 (03), 211–218. 10.4236/jssm.2016.93025

[B99] Yu Y. (2025). Towards a critical geography of aging: political economy, biopolitics, and care. Prog. Hum. Geogr. 49 (2), 164–181. 10.1177/03091325251321708

